# Supplementary material for: Dual-layer transposon repression in heads of Drosophila melanogaster
Source: RNA. 2018 Dec;24(12):1749–60. doi: 10.1261/rna.067173.118 (PMC6239173; doi:10.1261/rna.067173.118)
Supplement: Supplemental Material [file supp_24_12_1749__index.html]

Dual-layer transposon repression in heads of Drosophila melanogaster — Supplemental Material 

# Dual-layer transposon repression in heads of *Drosophila melanogaster*

## Supplemental Material

- Supplemental\_Material.pdf
