# Supplementary material for: Dual-layer transposon repression in heads of Drosophila melanogaster
Source: RNA. 2018 Dec;24(12):1749–60. doi: 10.1261/rna.067173.118 (PMC6239173; doi:10.1261/rna.067173.118)
Supplement: Supplemental Material [file supp_067173.118_Supplemental_Material.pdf]

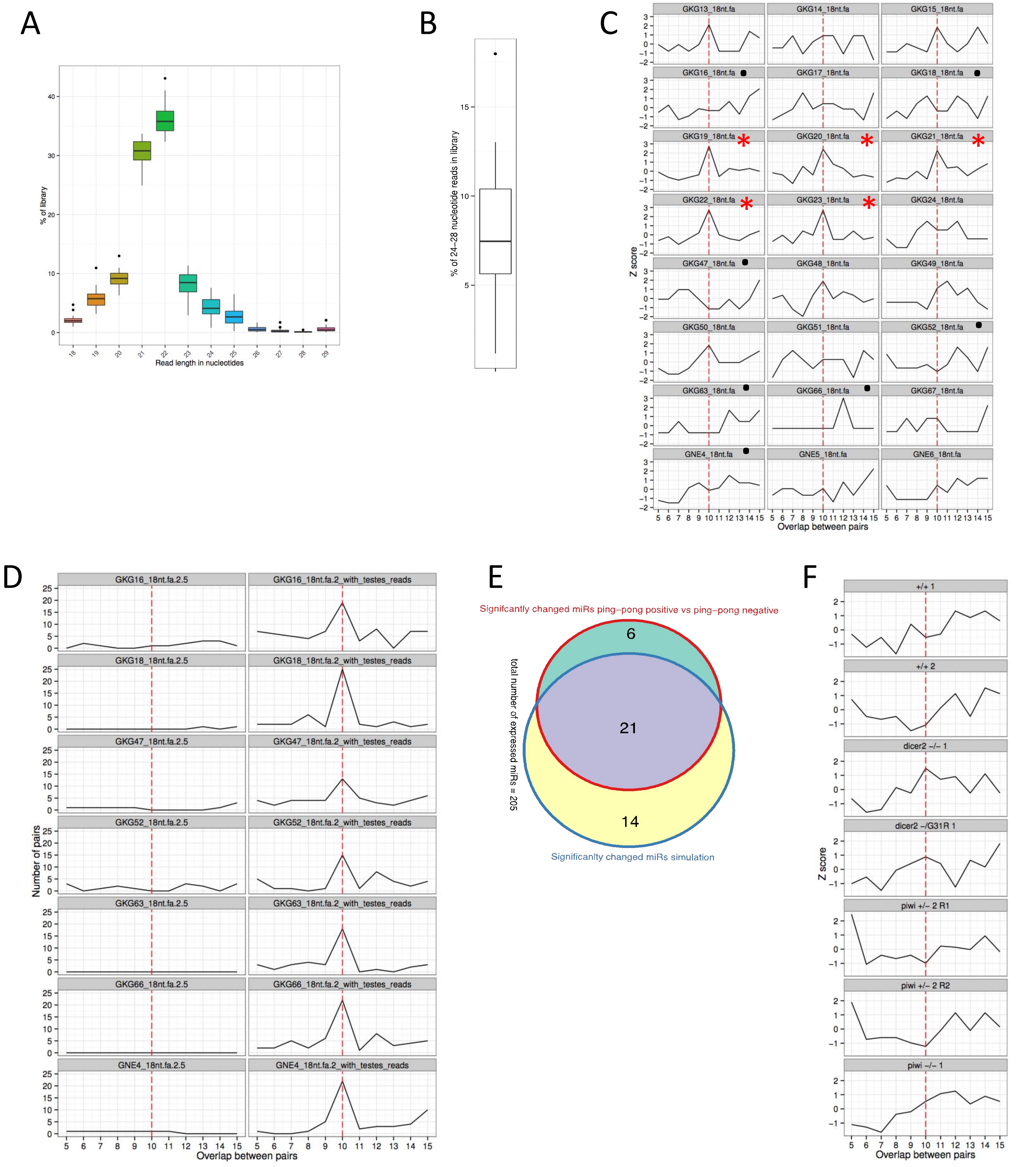

Supplemental Figure 1. Drosophila small RNA head libraries have variable amount of piRNA-sized reads and ping-pong signature in heads, which correlates with a contamination signature. (A) Boxplot showing the distribution of read lengths for the libraries analyzed in (C). (B) similar to (A), but for the fraction of 24-28 nucleotide length reads. (C) Ping-pong signature - tendency for small RNAs to overlap. The number of overlaps was transformed to Z scores to take into account the “cleanness” of the 10 nucleotide overlap as compared to other lengths of overlaps. Libraries with red asterisks were selected as ping-pong positive, and black dots indicate libraries selected as ping-pong negative libraries. (D) Ping-pong signature for 2.5*106 ping-pong negative libraries with (right group of panels) and without (left panels) the addition of 2% of a testis-library. Number of pairs are shown instead of Z scores, as some down-sampled libraries had 0 overlapping pairs. (E) Venn Diagram showing the overlap of differentially expressed miRNAs between ping-pong positive compared to ping-pong negative libraries (red circle) and ping-pong negative libraries compared to ping-pong negative libraries with the addition of 2% of a testis library (blue circle). (F) ping-pong signature for small RNA libraries (as in (C)) of indicated genotypes of heads closely dissected by hands.

A

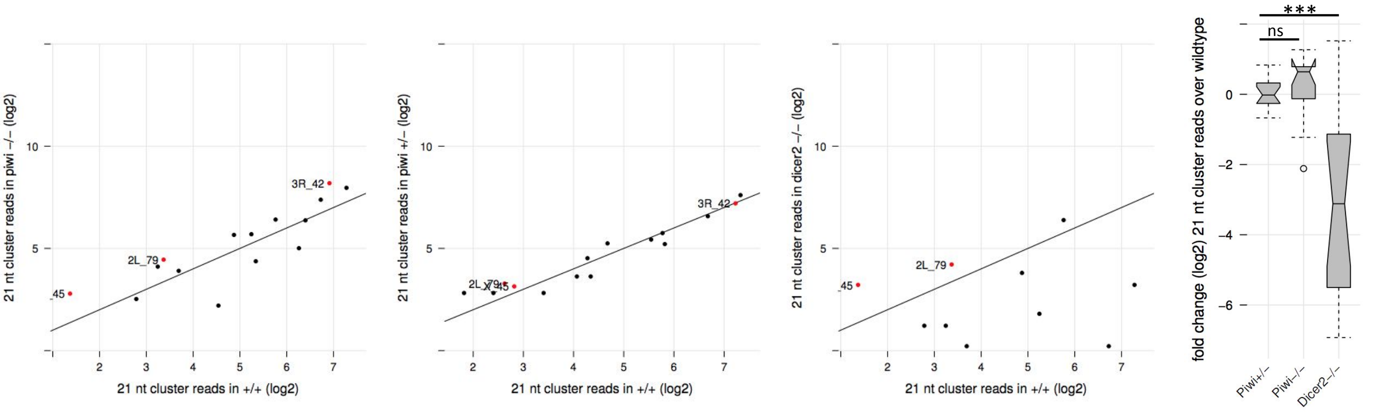

B

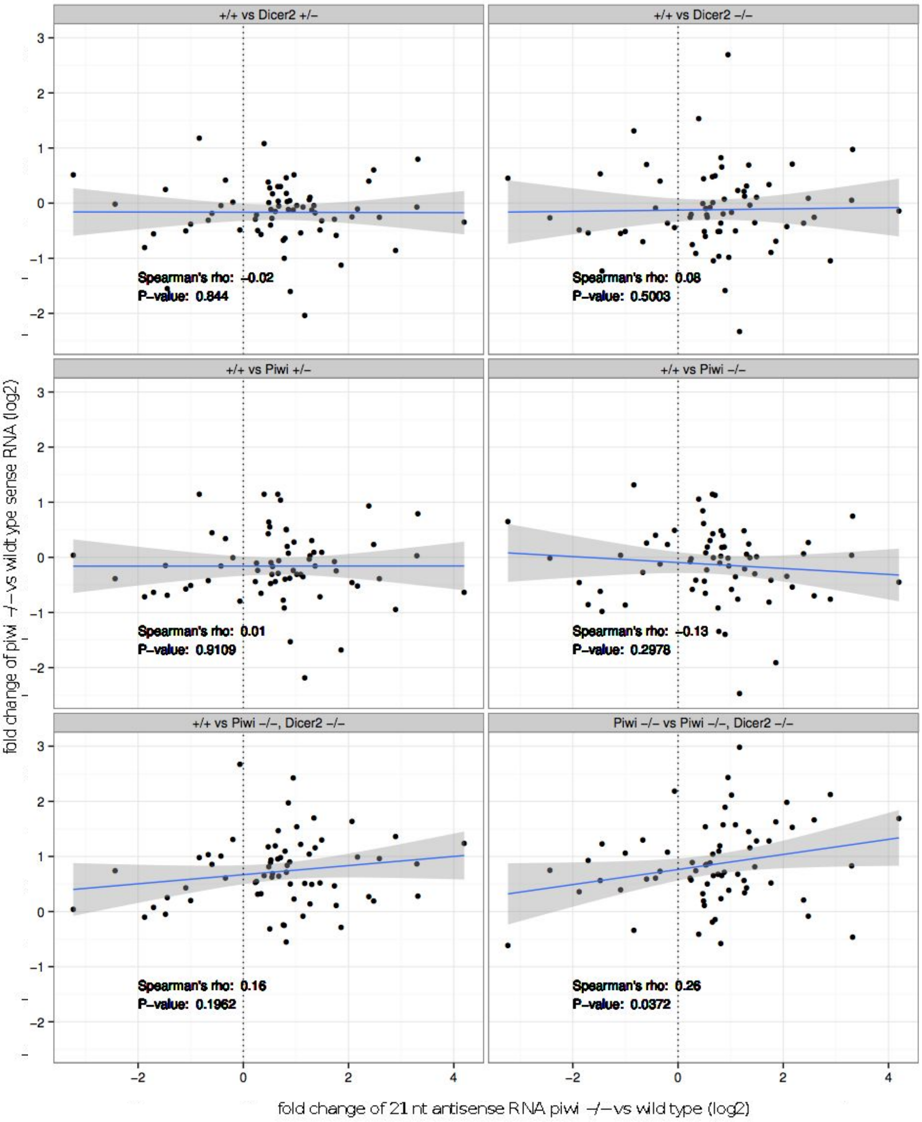

Supplemental Figure 2. (A) Scatterplots displaying the abundance of cluster-derived 21 nt reads in mutant (y-axis) and wild type (x-axis) heads. Red dots in the first panel indicate the cluster-specific 21 nt antisense reads that increased more than 2 fold in *piwi* -/- mutant heads. These dots are shown for comparison in the second and third panel. (A-right) Boxplots showing the distribution of 21 nt read fold changes (y-axis) between wild type and the indicated mutants (x-axis). Significance of differences between the distributions was assessed with Mann-Whitney U test. (B) Related to Figure 2E. Scatterplot displaying the correlation between log2 fold changes of 21 nt antisense RNA in *piwi* homozygous mutant heads compared to wild type heads on the x-axis and log2 fold changes of sense TE transcripts for the genotype comparisons indicated above each panel. All TE families that passed a threshold of on average five 21 nucleotide antisense reads over all small RNA libraries were analysed. The blue line is a fit produced by the lmfit function, and the grey area delimits the corresponding confidence interval.

Supplementary Table S1

Differential expression testing using EdgeR between ping-pong negative and ping-pong positive libraries (Sheet #1)

The top 10 differentially detected miRNA between ping-pong negative libraries and ping-pong negative libraries supplemented with 2% testicular reads (Sheet 2) are highlighted in red.
Original data at https://lbcd41.snv.jussieu.fr/artbio/u/marius-ged/h/compare-simulation-with-real-differences

Name	logFC	logCPM	LR	PValue	adj.p.value	Dispersion	Total reads
dme-mir-31b	2.57E+00	6.10E+00	6.44E+01	1.03E-15	2.25E-13	1.34E-01	3.86E+03
dme-mir-959	3.76E+00	3.61E+00	4.95E+01	1.98E-12	2.16E-10	3.19E-01	6.71E+02
dme-mir-991	2.51E+00	3.22E+00	3.81E+01	6.59E-10	4.79E-08	1.85E-01	4.96E+02
dme-mir-983-2	2.78E+00	2.65E+00	3.62E+01	1.75E-09	9.56E-08	2.18E-01	3.22E+02
dme-mir-961	2.99E+00	2.69E+00	3.44E+01	4.40E-09	1.92E-07	2.85E-01	3.38E+02
dme-mir-310	3.81E+00	5.06E-01	3.18E+01	1.68E-08	5.36E-07	1.80E-01	5.40E+01
dme-mir-963	3.28E+00	2.01E+00	3.18E+01	1.72E-08	5.36E-07	3.20E-01	1.95E+02
dme-mir-960	2.72E+00	4.18E+00	3.15E+01	2.04E-08	5.56E-07	2.97E-01	1.03E+03
dme-mir-985	3.86E+00	3.66E+00	2.99E+01	4.65E-08	1.13E-06	5.82E-01	6.96E+02
dme-mir-2494	4.26E+00	6.81E-02	2.79E+01	1.28E-07	2.65E-06	1.59E-01	3.20E+01
dme-mir-983-1	2.48E+00	2.54E+00	2.78E+01	1.34E-07	2.65E-06	2.32E-01	2.92E+02
dme-mir-982	1.88E+00	3.77E+00	2.63E+01	2.85E-07	5.00E-06	1.61E-01	7.42E+02
dme-mir-312	2.18E+00	3.18E+00	2.63E+01	2.98E-07	5.00E-06	2.09E-01	4.84E+02
dme-mir-iab-8	3.52E+00	5.98E-01	2.37E+01	1.12E-06	1.74E-05	3.05E-01	5.80E+01
dme-mir-375	1.21E+00	9.09E+00	2.27E+01	1.94E-06	2.57E-05	9.16E-02	3.04E+04
dme-mir-977	2.70E+00	3.42E+00	2.26E+01	1.97E-06	2.57E-05	4.01E-01	5.84E+02
dme-mir-976	2.92E+00	5.33E-01	2.26E+01	2.00E-06	2.57E-05	1.86E-01	5.40E+01
dme-mir-984	1.94E+00	2.56E+00	2.20E+01	2.73E-06	3.31E-05	1.86E-01	3.05E+02
dme-mir-956	3.35E+00	8.13E+00	2.06E+01	5.75E-06	6.59E-05	7.09E-01	1.58E+04
dme-mir-314	3.66E+00	7.04E+00	1.94E+01	1.04E-05	1.13E-04	8.74E-01	7.46E+03
dme-mir-962	2.67E+00	5.17E-01	1.84E+01	1.79E-05	1.86E-04	2.14E-01	5.20E+01
dme-mir-4966	2.82E+00	9.68E-02	1.74E+01	3.01E-05	2.92E-04	1.58E-01	3.30E+01
dme-mir-974	1.84E+00	1.61E+00	1.74E+01	3.08E-05	2.92E-04	1.70E-01	1.42E+02
dme-mir-1015	2.91E+00	3.39E-01	1.50E+01	1.06E-04	9.62E-04	3.74E-01	4.50E+01
dme-mir-311	2.16E+00	3.77E+00	1.48E+01	1.19E-04	1.04E-03	4.11E-01	7.50E+02
dme-mir-4914	2.90E+00	-2.59E-01	1.32E+01	2.84E-04	2.38E-03	1.62E-01	2.10E+01
dme-mir-973	2.03E+00	8.04E-01	1.09E+01	9.60E-04	7.75E-03	3.15E-01	6.90E+01
dme-mir-iab-4	1.57E+00	1.27E+00	1.01E+01	1.52E-03	1.18E-02	2.07E-01	1.05E+02
dme-mir-958	2.76E+00	3.83E+00	9.00E+00	2.69E-03	2.02E-02	1.12E+00	7.52E+02
dme-mir-964	1.51E+00	1.19E+00	8.60E+00	3.37E-03	2.45E-02	2.37E-01	1.00E+02
dme-mir-4983	-3.25E+00	-3.19E-01	8.34E+00	3.88E-03	2.73E-02	7.58E-01	1.90E+01
dme-mir-4939	2.81E+00	-5.69E-01	7.97E+00	4.75E-03	3.24E-02	3.28E-01	1.20E+01
dme-mir-12	5.09E-01	1.12E+01	7.47E+00	6.29E-03	4.16E-02	4.93E-02	1.31E+05
dme-mir-979	2.33E+00	-4.59E-01	7.15E+00	7.50E-03	4.81E-02	2.37E-01	1.50E+01
dme-mir-33	-7.46E-01	1.19E+01	6.91E+00	8.58E-03	5.35E-02	1.09E-01	2.04E+05
dme-mir-34	-4.93E-01	1.54E+01	6.69E+00	9.71E-03	5.88E-02	4.97E-02	2.29E+06
dme-mir-972	1.18E+00	2.38E+00	6.31E+00	1.20E-02	7.08E-02	2.61E-01	2.63E+02
dme-mir-31a	5.52E-01	1.19E+01	6.02E+00	1.42E-02	8.13E-02	7.21E-02	2.09E+05
dme-mir-10	5.94E-01	1.12E+01	5.90E+00	1.52E-02	8.32E-02	8.49E-02	1.29E+05
dme-mir-978	2.48E+00	-6.88E-01	5.89E+00	1.53E-02	8.32E-02	2.90E-01	9.00E+00
dme-mir-92a	-1.01E+00	6.43E+00	5.82E+00	1.59E-02	8.43E-02	2.31E-01	4.92E+03
dme-mir-4964	1.74E+00	-3.44E-01	5.78E+00	1.62E-02	8.43E-02	9.98E-02	1.80E+01
dme-mir-4972	2.73E+00	-9.28E-01	5.41E+00	2.00E-02	1.01E-01	3.22E-01	4.00E+00
dme-mir-966	-5.83E-01	5.34E+00	5.31E+00	2.13E-02	1.05E-01	8.08E-02	2.05E+03
dme-mir-100	-6.04E-01	9.61E+00	5.21E+00	2.25E-02	1.09E-01	9.50E-02	4.20E+04
dme-mir-184	6.53E-01	1.52E+01	4.97E+00	2.57E-02	1.22E-01	1.22E-01	2.03E+06
dme-mir-2b-1	-4.84E-01	1.10E+01	4.88E+00	2.72E-02	1.26E-01	6.57E-02	1.09E+05
dme-mir-1014	1.53E+00	9.49E-01	4.85E+00	2.77E-02	1.26E-01	5.28E-01	8.00E+01
dme-mir-303	2.26E+00	-7.32E-01	4.75E+00	2.92E-02	1.30E-01	2.90E-01	8.00E+00
dme-mir-996	4.22E-01	1.23E+01	4.69E+00	3.04E-02	1.32E-01	5.40E-02	2.76E+05
dme-mir-87	-6.01E-01	8.41E+00	4.63E+00	3.13E-02	1.34E-01	1.05E-01	1.81E+04
dme-mir-997	1.82E+00	-4.22E-01	4.12E+00	4.25E-02	1.78E-01	4.31E-01	1.60E+01
dme-mir-2b-2	-4.09E-01	1.13E+01	4.07E+00	4.38E-02	1.80E-01	5.66E-02	1.34E+05
dme-mir-4910	-9.95E-01	1.78E+00	3.89E+00	4.86E-02	1.96E-01	2.63E-01	1.60E+02
dme-mir-1	-7.10E-01	1.68E+01	3.85E+00	4.99E-02	1.98E-01	1.77E-01	5.84E+06
dme-mir-190	-4.45E-01	1.06E+01	3.75E+00	5.28E-02	2.06E-01	7.23E-02	8.24E+04
dme-mir-6-3	1.39E+00	-1.52E-01	3.70E+00	5.45E-02	2.08E-01	2.70E-01	2.40E+01
dme-mir-283	5.51E-01	9.47E+00	3.61E+00	5.75E-02	2.16E-01	1.19E-01	3.88E+04
dme-mir-210	5.02E-01	1.35E+01	3.44E+00	6.36E-02	2.32E-01	1.04E-01	6.40E+05
dme-mir-957	5.30E-01	1.23E+01	3.43E+00	6.39E-02	2.32E-01	1.17E-01	2.80E+05
dme-mir-975	1.39E+00	-4.50E-01	3.24E+00	7.17E-02	2.56E-01	1.46E-01	1.50E+01
dme-mir-315	5.52E-01	1.07E+01	3.21E+00	7.30E-02	2.57E-01	1.35E-01	9.92E+04

dme-mir-281-2	3.81E-01	1.02E+01	3.14E+00	7.62E-02	2.64E-01	6.53E-02	6.46E+04
dme-mir-5	9.99E-01	5.34E+00	3.08E+00	7.95E-02	2.71E-01	4.57E-01	2.12E+03
dme-mir-2280	-1.59E+00	-5.05E-01	3.02E+00	8.23E-02	2.76E-01	3.65E-01	1.30E+01
dme-mir-927	-3.54E-01	1.10E+01	2.90E+00	8.85E-02	2.92E-01	5.96E-02	1.14E+05
dme-mir-2a-1	-4.10E-01	9.55E+00	2.80E+00	9.44E-02	3.07E-01	8.22E-02	4.01E+04
dme-mir-263b	-3.74E-01	1.21E+01	2.75E+00	9.72E-02	3.12E-01	7.02E-02	2.47E+05
dme-mir-981	3.73E-01	9.80E+00	2.68E+00	1.02E-01	3.17E-01	7.35E-02	4.79E+04
dme-mir-988	-3.99E-01	1.01E+01	2.68E+00	1.02E-01	3.17E-01	8.14E-02	5.97E+04
dme-mir-1000	3.85E-01	1.15E+01	2.55E+00	1.10E-01	3.38E-01	8.23E-02	1.54E+05
dme-mir-3645	-1.69E+00	-8.26E-01	2.52E+00	1.12E-01	3.40E-01	1.84E-01	6.00E+00
dme-mir-4945	-2.16E+00	2.58E-01	2.48E+00	1.16E-01	3.45E-01	2.04E+00	4.20E+01
dme-mir-305	3.81E-01	1.20E+01	2.42E+00	1.20E-01	3.53E-01	8.52E-02	2.18E+05
dme-mir-276a	-2.76E-01	1.69E+01	2.38E+00	1.23E-01	3.58E-01	4.43E-02	6.58E+06
dme-mir-2501	1.40E+00	-7.44E-01	2.11E+00	1.47E-01	4.20E-01	2.22E-01	8.00E+00
dme-mir-281-1	3.67E-01	8.21E+00	2.05E+00	1.52E-01	4.20E-01	9.24E-02	1.66E+04
dme-mir-124	3.30E-01	9.39E+00	2.05E+00	1.52E-01	4.20E-01	7.49E-02	3.57E+04
dme-mir-318	-5.56E-01	2.50E+00	2.02E+00	1.56E-01	4.20E-01	1.65E-01	2.74E+02
dme-mir-1011	7.25E-01	3.72E+00	2.01E+00	1.56E-01	4.20E-01	3.55E-01	6.99E+02
dme-mir-954	3.68E-01	6.28E+00	2.01E+00	1.56E-01	4.20E-01	9.23E-02	4.24E+03
dme-mir-3644	1.57E+00	-8.77E-01	1.97E+00	1.60E-01	4.26E-01	3.45E-01	5.00E+00
dme-mir-4982	1.53E+00	-8.82E-01	1.92E+00	1.66E-01	4.37E-01	3.06E-01	5.00E+00
dme-mir-995	3.31E-01	1.02E+01	1.87E+00	1.71E-01	4.44E-01	8.28E-02	6.38E+04
dme-mir-1010	3.10E-01	1.05E+01	1.86E+00	1.73E-01	4.44E-01	7.30E-02	7.63E+04
dme-mir-307b	-7.45E-01	7.91E-01	1.80E+00	1.80E-01	4.56E-01	2.49E-01	6.70E+01
dme-mir-980	4.13E-01	3.31E+00	1.65E+00	1.99E-01	4.99E-01	1.20E-01	4.99E+02
dme-mir-4973	6.16E-01	1.04E+00	1.60E+00	2.06E-01	5.09E-01	1.96E-01	8.70E+01
dme-mir-313	7.52E-01	7.66E-01	1.55E+00	2.13E-01	5.21E-01	3.46E-01	6.80E+01
dme-mir-317	-2.29E-01	1.78E+01	1.43E+00	2.32E-01	5.60E-01	5.11E-02	1.16E+07
dme-mir-282	3.21E-01	8.97E+00	1.42E+00	2.34E-01	5.60E-01	1.02E-01	2.68E+04
dme-mir-4957	7.00E-01	-6.33E-03	1.39E+00	2.39E-01	5.66E-01	1.25E-01	2.90E+01
dme-mir-992	1.16E+00	-7.86E-01	1.36E+00	2.43E-01	5.68E-01	2.40E-01	7.00E+00
dme-mir-2c	3.58E-01	6.14E+00	1.35E+00	2.45E-01	5.68E-01	1.31E-01	3.93E+03
dme-mir-1005	3.78E-01	4.93E+00	1.29E+00	2.56E-01	5.81E-01	1.49E-01	1.65E+03
dme-mir-4	7.52E-01	3.15E+00	1.26E+00	2.61E-01	5.81E-01	6.11E-01	4.42E+02
dme-mir-3	-8.78E-01	3.95E+00	1.25E+00	2.63E-01	5.81E-01	8.09E-01	8.57E+02
dme-mir-4951	2.90E-01	5.55E+00	1.21E+00	2.71E-01	5.81E-01	9.32E-02	2.58E+03
dme-mir-11	-2.50E-01	1.23E+01	1.21E+00	2.71E-01	5.81E-01	7.14E-02	2.72E+05
dme-mir-4943	5.53E-01	8.37E-01	1.20E+00	2.73E-01	5.81E-01	1.98E-01	7.30E+01
dme-mir-286	-4.85E-01	3.78E+00	1.20E+00	2.74E-01	5.81E-01	2.52E-01	7.19E+02
dme-mir-970	2.53E-01	1.16E+01	1.19E+00	2.75E-01	5.81E-01	7.57E-02	1.68E+05
dme-mir-955	6.09E-01	9.02E-01	1.19E+00	2.75E-01	5.81E-01	2.82E-01	7.60E+01
dme-mir-2497	7.93E-01	9.17E-01	1.17E+00	2.80E-01	5.81E-01	6.24E-01	7.80E+01
dme-mir-987	2.41E-01	1.35E+01	1.15E+00	2.83E-01	5.81E-01	7.16E-02	6.61E+05
dme-mir-2a-2	-2.13E-01	1.04E+01	1.14E+00	2.85E-01	5.81E-01	5.51E-02	7.35E+04
dme-mir-969	-3.14E-01	4.95E+00	1.13E+00	2.87E-01	5.81E-01	1.13E-01	1.68E+03
dme-mir-971	2.97E-01	5.48E+00	1.13E+00	2.88E-01	5.81E-01	1.05E-01	2.40E+03
dme-mir-1003	-3.32E-01	6.49E+00	1.11E+00	2.93E-01	5.85E-01	1.34E-01	4.63E+03
dme-mir-998	-2.40E-01	8.81E+00	1.08E+00	2.99E-01	5.92E-01	7.35E-02	2.41E+04
dme-mir-4974	8.99E-01	-6.88E-01	1.04E+00	3.08E-01	6.06E-01	1.37E-01	9.00E+00
dme-mir-4913	4.98E-01	8.04E-01	9.99E-01	3.17E-01	6.18E-01	1.81E-01	6.90E+01
dme-mir-193	2.17E-01	1.10E+01	9.52E-01	3.29E-01	6.35E-01	6.96E-02	1.10E+05
dme-mir-4919	3.75E-01	2.53E+00	9.32E-01	3.34E-01	6.40E-01	1.69E-01	2.80E+02
dme-mir-986	-2.53E-01	1.18E+01	8.95E-01	3.44E-01	6.52E-01	9.87E-02	1.86E+05
dme-mir-278	-1.72E-01	1.26E+01	8.73E-01	3.50E-01	6.58E-01	4.73E-02	3.49E+05
dme-mir-1008	3.08E-01	4.27E+00	8.50E-01	3.57E-01	6.64E-01	1.45E-01	9.93E+02
dme-mir-4969	-3.44E-01	3.58E+00	8.00E-01	3.71E-01	6.78E-01	1.84E-01	6.27E+02
dme-mir-2498	8.96E-01	-8.32E-01	7.87E-01	3.75E-01	6.78E-01	1.82E-01	6.00E+00
dme-mir-4981	4.81E-01	7.08E-01	7.83E-01	3.76E-01	6.78E-01	2.33E-01	6.30E+01
dme-mir-307a	1.98E-01	1.10E+01	7.82E-01	3.76E-01	6.78E-01	7.03E-02	1.05E+05
dme-mir-1004	-2.32E-01	7.57E+00	7.53E-01	3.85E-01	6.80E-01	9.78E-02	1.03E+04
dme-bantam	-2.16E-01	1.45E+01	7.45E-01	3.88E-01	6.80E-01	8.70E-02	1.26E+06
dme-mir-967	3.74E-01	1.55E+00	7.42E-01	3.89E-01	6.80E-01	1.75E-01	1.34E+02
dme-mir-284	-1.81E-01	1.18E+01	7.35E-01	3.91E-01	6.80E-01	6.19E-02	1.96E+05
dme-mir-4961	-4.55E-01	5.59E-01	7.29E-01	3.93E-01	6.80E-01	1.80E-01	5.30E+01
dme-mir-2495	8.55E-01	-8.32E-01	7.09E-01	4.00E-01	6.86E-01	2.39E-01	6.00E+00
dme-mir-275	2.27E-01	1.05E+01	6.85E-01	4.08E-01	6.95E-01	1.06E-01	8.10E+04
dme-mir-1013	2.61E-01	5.71E+00	6.56E-01	4.18E-01	7.06E-01	1.42E-01	2.94E+03
dme-mir-277	-1.40E-01	1.42E+01	6.38E-01	4.25E-01	7.12E-01	4.31E-02	9.83E+05

dme-mir-2489	3.27E-01	1.88E+00	6.19E-01	4.31E-01	7.18E-01	1.73E-01	1.75E+02
dme-mir-4956	-5.27E-01	-3.45E-01	5.64E-01	4.53E-01	7.48E-01	1.31E-01	1.80E+01
dme-mir-4940	-5.57E-01	2.02E+00	5.45E-01	4.60E-01	7.51E-01	7.18E-01	1.96E+02
dme-mir-999	-1.31E-01	1.46E+01	5.42E-01	4.62E-01	7.51E-01	4.38E-02	1.36E+06
dme-mir-13b-2	1.91E-01	1.22E+01	5.28E-01	4.68E-01	7.54E-01	9.71E-02	2.64E+05
dme-mir-3641	-6.14E-01	-5.35E-01	5.11E-01	4.75E-01	7.54E-01	3.06E-01	1.30E+01
dme-mir-276b	-1.20E-01	1.37E+01	5.03E-01	4.78E-01	7.54E-01	4.01E-02	7.07E+05
dme-mir-13b-1	1.87E-01	1.22E+01	5.01E-01	4.79E-01	7.54E-01	9.83E-02	2.64E+05
dme-mir-4975	4.03E-01	2.37E-01	4.94E-01	4.82E-01	7.54E-01	1.81E-01	3.90E+01
dme-mir-8	1.67E-01	1.51E+01	4.88E-01	4.85E-01	7.54E-01	8.06E-02	1.87E+06
dme-mir-4909	4.47E-01	-1.06E-01	4.72E-01	4.92E-01	7.54E-01	1.78E-01	2.50E+01
dme-mir-932	-1.32E-01	1.06E+01	4.60E-01	4.98E-01	7.54E-01	5.26E-02	8.40E+04
dme-mir-1002	5.00E-01	-1.50E-01	4.59E-01	4.98E-01	7.54E-01	3.48E-01	2.40E+01
dme-mir-1009	-2.10E-01	4.83E+00	4.57E-01	4.99E-01	7.54E-01	1.26E-01	1.57E+03
dme-mir-219	2.48E-01	5.24E+00	4.51E-01	5.02E-01	7.54E-01	1.86E-01	2.01E+03
dme-mir-2535b	-1.85E-01	4.26E+00	4.31E-01	5.12E-01	7.64E-01	9.73E-02	9.91E+02
dme-mir-263a	1.47E-01	1.44E+01	4.15E-01	5.19E-01	7.66E-01	7.33E-02	1.17E+06
dme-mir-9a	-1.28E-01	1.42E+01	4.07E-01	5.23E-01	7.66E-01	5.64E-02	9.34E+05
dme-mir-4912	7.58E-01	-9.80E-01	4.07E-01	5.24E-01	7.66E-01	3.22E-01	3.00E+00
dme-mir-306	1.27E-01	1.18E+01	3.42E-01	5.58E-01	8.07E-01	6.67E-02	2.02E+05
dme-mir-308	2.41E-01	6.60E+00	3.39E-01	5.60E-01	8.07E-01	2.40E-01	5.15E+03
dme-mir-4984	3.31E-01	5.81E-01	3.31E-01	5.65E-01	8.07E-01	2.60E-01	5.50E+01
dme-mir-4952	1.77E-01	3.87E+00	3.25E-01	5.68E-01	8.07E-01	1.18E-01	7.62E+02
dme-mir-994	-2.88E-01	1.25E+00	3.17E-01	5.74E-01	8.07E-01	2.47E-01	1.01E+02
dme-mir-1012	1.31E-01	9.90E+00	3.16E-01	5.74E-01	8.07E-01	7.64E-02	4.98E+04
dme-mir-2493	-3.23E-01	1.31E-01	3.05E-01	5.81E-01	8.09E-01	1.56E-01	3.40E+01
dme-mir-1016	-1.99E-01	2.49E+00	3.02E-01	5.83E-01	8.09E-01	1.37E-01	2.70E+02
dme-mir-4986	-4.30E-01	-4.50E-01	2.95E-01	5.87E-01	8.10E-01	2.53E-01	1.50E+01
dme-let-7	8.95E-02	1.53E+01	2.66E-01	6.06E-01	8.26E-01	4.23E-02	2.08E+06
dme-mir-4946	-6.07E-01	-9.76E-01	2.65E-01	6.06E-01	8.26E-01	3.22E-01	3.00E+00
dme-mir-4918	-4.22E-01	-6.46E-01	2.47E-01	6.19E-01	8.33E-01	1.36E-01	1.00E+01
dme-mir-133	-9.74E-02	1.21E+01	2.47E-01	6.19E-01	8.33E-01	5.35E-02	2.35E+05
dme-mir-2496	-4.32E-01	-2.42E-01	2.42E-01	6.23E-01	8.33E-01	6.31E-01	2.10E+01
dme-mir-4944	-5.50E-01	-9.84E-01	2.32E-01	6.30E-01	8.38E-01	3.22E-01	3.00E+00
dme-mir-7	-1.29E-01	1.53E+01	2.20E-01	6.39E-01	8.44E-01	1.05E-01	2.34E+06
dme-mir-6-2	4.18E-01	3.03E-02	1.97E-01	6.57E-01	8.55E-01	9.37E-01	3.00E+01
dme-mir-13a	-1.34E-01	8.06E+00	1.96E-01	6.58E-01	8.55E-01	1.28E-01	1.41E+04
dme-mir-274	9.96E-02	1.47E+01	1.93E-01	6.60E-01	8.55E-01	7.22E-02	1.40E+06
dme-mir-316	1.13E-01	6.40E+00	1.89E-01	6.63E-01	8.55E-01	9.21E-02	4.54E+03
dme-mir-252	-7.78E-02	1.38E+01	1.85E-01	6.67E-01	8.55E-01	4.57E-02	8.06E+05
dme-mir-6-1	-3.16E-01	-3.52E-01	1.74E-01	6.77E-01	8.59E-01	3.17E-01	1.80E+01
dme-mir-2283	-2.30E-01	1.89E-01	1.70E-01	6.80E-01	8.59E-01	1.45E-01	3.70E+01
dme-mir-4987	-4.21E-01	-7.37E-01	1.68E-01	6.82E-01	8.59E-01	6.70E-01	8.00E+00
dme-mir-4947	-2.93E-01	-4.07E-01	1.46E-01	7.02E-01	8.77E-01	2.10E-01	1.60E+01
dme-mir-79	-8.78E-02	7.90E+00	1.42E-01	7.06E-01	8.77E-01	7.46E-02	1.27E+04
dme-mir-4968	-1.46E-01	2.07E+00	1.41E-01	7.08E-01	8.77E-01	1.50E-01	2.02E+02
dme-mir-990	-1.06E-01	5.01E+00	1.25E-01	7.23E-01	8.91E-01	1.18E-01	1.75E+03
dme-mir-4949	-2.18E-01	-9.14E-02	1.19E-01	7.30E-01	8.94E-01	1.40E-01	2.60E+01
dme-mir-4953	-3.83E-01	-8.72E-01	1.13E-01	7.37E-01	8.98E-01	6.60E-01	5.00E+00
dme-mir-2492	-3.48E-01	-8.77E-01	1.07E-01	7.44E-01	9.01E-01	3.76E-01	5.00E+00
dme-mir-4971	-2.44E-01	-4.11E-01	9.37E-02	7.60E-01	9.08E-01	3.01E-01	1.60E+01
dme-mir-2282	-2.32E-01	-3.09E-01	9.24E-02	7.61E-01	9.08E-01	3.36E-01	1.90E+01
dme-mir-309	3.03E-01	2.29E-01	8.82E-02	7.66E-01	9.08E-01	1.23E+00	3.90E+01
dme-mir-4980	-2.95E-01	-7.85E-01	8.77E-02	7.67E-01	9.08E-01	2.83E-01	7.00E+00
dme-mir-279	7.32E-02	1.05E+01	8.50E-02	7.71E-01	9.08E-01	8.84E-02	7.55E+04
dme-mir-4958	1.36E-01	1.48E+00	8.15E-02	7.75E-01	9.09E-01	2.20E-01	1.25E+02

http://lbcd41.snv.jussieu.fr/artbio/u/marius-ged/h/compare-simulation-with-real-differences

Differential expression analysis between ping-pong negative libraries and ping-pong negative libraries supplemented with 2% testes reads (sheet#2)

The 19 differentially detected miRNA between ping-pong negative libraries and ping-pong positive libraries (Sheet 1) are highlighted in red

The remaining 8 miRNAs are very lowly expressed and were not sampled during the downsampling process.

Name	logFC	logCPM	LR	PValue	adj.p.value	Dispersion	totreads
dme-mir-964	6.30E+00	5.74E+00	1.18E+03	3.54E-258	7.29E-256	4.31E-04	9.87E+02
dme-mir-959	5.61E+00	5.57E+00	9.99E+02	3.01E-219	3.10E-217	3.89E-04	8.76E+02
dme-mir-962	8.52E+00	5.28E+00	9.31E+02	1.79E-204	1.23E-202	2.54E-04	7.11E+02
dme-mir-984	5.42E+00	5.46E+00	9.18E+02	1.08E-201	5.54E-200	3.54E-04	8.15E+02
dme-mir-985	4.96E+00	5.10E+00	6.78E+02	1.44E-149	5.94E-148	2.41E-04	6.28E+02
dme-mir-974	5.43E+00	4.66E+00	5.17E+02	1.63E-114	5.60E-113	1.42E-04	4.52E+02
dme-mir-991	4.29E+00	4.83E+00	4.97E+02	4.35E-110	1.28E-108	1.32E-04	5.10E+02
dme-mir-960	3.11E+00	4.85E+00	3.69E+02	2.75E-82	7.09E-81	1.73E-04	5.24E+02
dme-mir-961	4.48E+00	4.16E+00	3.17E+02	6.53E-71	1.49E-69	6.58E-05	3.11E+02
dme-mir-312	3.19E+00	4.22E+00	2.37E+02	1.41E-53	2.90E-52	1.02E-04	3.24E+02
dme-mir-997	5.58E+00	3.55E+00	2.31E+02	4.06E-52	7.61E-51	3.34E-07	1.95E+02
dme-mir-976	7.32E+00	3.15E+00	1.90E+02	2.95E-43	5.06E-42	7.77E-07	1.41E+02
dme-mir-977	2.84E+00	3.81E+00	1.54E+02	2.45E-35	3.88E-34	7.28E-06	2.41E+02
dme-mir-31b	1.35E+00	5.36E+00	1.46E+02	1.37E-33	2.02E-32	3.28E-04	7.61E+02
dme-mir-978	6.69E+00	2.67E+00	1.23E+02	1.26E-28	1.73E-27	1.12E-07	9.20E+01
dme-mir-989	2.63E+00	3.57E+00	1.15E+02	9.46E-27	1.22E-25	3.54E-07	1.98E+02
dme-mir-963	3.46E+00	2.97E+00	9.96E+01	1.85E-23	2.24E-22	5.76E-09	1.21E+02
dme-mir-983-2	2.46E+00	2.87E+00	5.94E+01	1.26E-14	1.45E-13	2.68E-08	1.11E+02
dme-mir-992	5.59E+00	1.89E+00	5.64E+01	5.79E-14	6.28E-13	6.19E-04	4.20E+01
dme-mir-313	2.68E+00	2.52E+00	4.96E+01	1.92E-12	1.92E-11	1.40E-04	8.10E+01
dme-mir-982	1.39E+00	3.81E+00	4.95E+01	1.96E-12	1.92E-11	2.40E-05	2.37E+02
dme-mir-983-1	2.06E+00	2.85E+00	4.48E+01	2.14E-11	2.00E-10	3.17E-08	1.09E+02
dme-mir-303	5.06E+00	1.59E+00	3.88E+01	4.65E-10	4.16E-09	1.54E-06	2.90E+01
dme-mir-318	1.18E+00	3.60E+00	3.15E+01	1.98E-08	1.70E-07	3.94E-07	2.03E+02
dme-mir-4966	3.91E+00	1.59E+00	3.04E+01	3.43E-08	2.83E-07	9.22E-04	2.90E+01
dme-mir-310	3.80E+00	1.54E+00	2.79E+01	1.29E-07	1.03E-06	1.65E-06	2.70E+01
dme-mir-311	1.19E+00	3.23E+00	2.40E+01	9.81E-07	7.49E-06	7.38E-08	1.51E+02
dme-mir-973	2.29E+00	1.96E+00	2.32E+01	1.44E-06	1.06E-05	5.31E-04	4.60E+01
dme-mir-979	4.25E+00	1.22E+00	2.17E+01	3.23E-06	2.29E-05	2.42E-06	1.60E+01
dme-mir-375	2.10E-01	8.56E+00	1.82E+01	2.04E-05	1.40E-04	2.05E-03	7.13E+03
dme-mir-316	3.18E-01	6.43E+00	1.81E+01	2.12E-05	1.41E-04	6.00E-04	1.61E+03
dme-mir-79	1.89E-01	8.04E+00	1.67E+01	4.42E-05	2.84E-04	7.49E-04	5.02E+03
dme-mir-2498	3.32E+00	9.24E-01	1.08E+01	1.03E-03	6.42E-03	3.09E-04	8.00E+00
dme-mir-999	-9.88E-02	1.46E+01	1.07E+01	1.07E-03	6.51E-03	1.50E-03	4.71E+05
dme-mir-278	-9.33E-02	1.26E+01	9.90E+00	1.65E-03	9.72E-03	1.36E-03	1.21E+05
dme-mir-305	-9.50E-02	1.18E+01	9.59E+00	1.96E-03	1.12E-02	1.35E-03	6.57E+04
dme-mir-274	-9.17E-02	1.46E+01	9.48E+00	2.08E-03	1.16E-02	1.46E-03	4.66E+05
dme-mir-193	-1.03E-01	1.08E+01	9.38E+00	2.20E-03	1.18E-02	1.45E-03	3.39E+04
dme-mir-987	-9.66E-02	1.34E+01	9.35E+00	2.23E-03	1.18E-02	1.60E-03	1.96E+05
dme-mir-317	-9.33E-02	1.79E+01	9.09E+00	2.57E-03	1.32E-02	1.61E-03	4.46E+06
dme-mir-184	-8.90E-02	1.48E+01	9.05E+00	2.63E-03	1.32E-02	1.44E-03	5.38E+05
dme-mir-986	-9.00E-02	1.19E+01	8.98E+00	2.74E-03	1.32E-02	1.30E-03	6.93E+04
dme-mir-981	-1.02E-01	9.60E+00	8.95E+00	2.77E-03	1.32E-02	9.53E-04	1.47E+04
dme-mir-927	-9.84E-02	1.11E+01	8.92E+00	2.82E-03	1.32E-02	1.47E-03	4.13E+04
dme-mir-276a	-9.09E-02	1.70E+01	8.87E+00	2.89E-03	1.33E-02	1.56E-03	2.49E+06
dme-mir-125	-8.77E-02	1.44E+01	8.54E+00	3.48E-03	1.56E-02	1.48E-03	4.20E+05
dme-mir-7	-9.07E-02	1.52E+01	8.44E+00	3.68E-03	1.60E-02	1.62E-03	7.26E+05
dme-mir-14	-8.94E-02	1.40E+01	8.40E+00	3.76E-03	1.60E-02	1.55E-03	3.14E+05
dme-mir-1	-8.95E-02	1.70E+01	8.38E+00	3.80E-03	1.60E-02	1.60E-03	2.34E+06
dme-mir-210	-8.84E-02	1.33E+01	8.26E+00	4.06E-03	1.67E-02	1.49E-03	1.90E+05
dme-mir-252	-8.93E-02	1.38E+01	8.12E+00	4.38E-03	1.77E-02	1.60E-03	2.72E+05
dme-mir-957	-8.10E-02	1.20E+01	8.01E+00	4.66E-03	1.85E-02	1.15E-03	7.83E+04
dme-mir-9a	-8.93E-02	1.42E+01	7.89E+00	4.97E-03	1.93E-02	1.66E-03	3.46E+05
dme-mir-284	-8.95E-02	1.19E+01	7.79E+00	5.25E-03	1.97E-02	1.51E-03	7.21E+04
dme-mir-277	-8.73E-02	1.42E+01	7.79E+00	5.27E-03	1.97E-02	1.61E-03	3.53E+05
dme-mir-190	-9.89E-02	1.07E+01	7.74E+00	5.39E-03	1.98E-02	1.64E-03	3.12E+04

dme-mir-13b-1	-7.63E-02	1.21E+01	7.65E+00	5.67E-03	2.05E-02	1.09E-03	8.48E+04
dme-let-7	-8.53E-02	1.52E+01	7.60E+00	5.85E-03	2.07E-02	1.59E-03	7.17E+05
dme-mir-993	-1.09E-01	8.82E+00	7.57E+00	5.94E-03	2.07E-02	9.64E-04	8.52E+03
dme-mir-100	-9.13E-02	9.76E+00	7.52E+00	6.11E-03	2.10E-02	9.55E-04	1.66E+04
dme-mir-276b	-8.39E-02	1.37E+01	7.46E+00	6.32E-03	2.14E-02	1.53E-03	2.61E+05
dme-mir-34	-8.14E-02	1.56E+01	7.43E+00	6.43E-03	2.14E-02	1.48E-03	9.19E+05
dme-mir-970	-9.45E-02	1.15E+01	7.39E+00	6.56E-03	2.15E-02	1.74E-03	5.50E+04
dme-mir-133	-8.33E-02	1.21E+01	7.28E+00	6.99E-03	2.25E-02	1.42E-03	8.46E+04
dme-mir-315	-8.75E-02	1.04E+01	7.08E+00	7.80E-03	2.47E-02	1.19E-03	2.54E+04
dme-mir-980	5.25E-01	3.63E+00	6.81E+00	9.04E-03	2.82E-02	4.31E-07	2.07E+02
dme-mir-1000	-8.18E-02	1.13E+01	6.59E+00	1.02E-02	3.15E-02	1.36E-03	4.80E+04
dme-mir-307a	-8.36E-02	1.08E+01	6.50E+00	1.08E-02	3.26E-02	1.37E-03	3.49E+04
dme-mir-932	-9.28E-02	1.06E+01	6.34E+00	1.18E-02	3.53E-02	1.80E-03	2.93E+04
dme-mir-263b	-8.50E-02	1.22E+01	6.24E+00	1.25E-02	3.68E-02	1.78E-03	8.86E+04
dme-mir-11	-7.76E-02	1.23E+01	6.09E+00	1.36E-02	3.94E-02	1.50E-03	1.00E+05
dme-mir-33	-8.24E-02	1.22E+01	5.94E+00	1.48E-02	4.19E-02	1.72E-03	8.93E+04
dme-mir-306	-7.81E-02	1.17E+01	5.92E+00	1.49E-02	4.19E-02	1.50E-03	6.62E+04
dme-mir-263a	-7.38E-02	1.43E+01	5.91E+00	1.50E-02	4.19E-02	1.51E-03	3.74E+05
dme-mir-998	-1.10E-01	8.84E+00	5.83E+00	1.58E-02	4.34E-02	1.79E-03	8.54E+03
dme-mir-995	-7.61E-02	1.01E+01	5.68E+00	1.71E-02	4.64E-02	9.69E-04	2.02E+04
dme-mir-1010	-8.20E-02	1.03E+01	5.58E+00	1.82E-02	4.86E-02	1.39E-03	2.38E+04
dme-mir-279	-8.01E-02	1.04E+01	5.16E+00	2.31E-02	6.10E-02	1.49E-03	2.66E+04
dme-mir-996	-7.55E-02	1.21E+01	5.04E+00	2.48E-02	6.48E-02	1.72E-03	8.25E+04
dme-mir-4958	-1.11E+00	1.66E+00	4.96E+00	2.59E-02	6.67E-02	7.99E-04	3.20E+01
dme-mir-975	2.03E+00	9.25E-01	4.81E+00	2.82E-02	7.18E-02	3.15E-06	8.00E+00
dme-mir-314	2.86E-01	4.70E+00	4.74E+00	2.95E-02	7.32E-02	1.87E-04	4.88E+02
dme-mir-1006	-1.38E-01	6.98E+00	4.74E+00	2.95E-02	7.32E-02	8.30E-04	2.38E+03
dme-mir-285	-7.92E-02	1.01E+01	4.54E+00	3.31E-02	8.12E-02	1.63E-03	2.18E+04
dme-mir-988	-7.89E-02	1.02E+01	4.43E+00	3.54E-02	8.57E-02	1.71E-03	2.21E+04
dme-mir-283	-7.89E-02	9.11E+00	4.40E+00	3.60E-02	8.62E-02	9.57E-04	1.02E+04
dme-mir-1001	-1.02E-01	7.90E+00	4.31E+00	3.78E-02	8.95E-02	9.61E-04	4.49E+03
dme-mir-124	-7.93E-02	9.21E+00	4.17E+00	4.11E-02	9.63E-02	1.22E-03	1.12E+04
dme-mir-1012	-7.28E-02	9.82E+00	4.11E+00	4.27E-02	9.88E-02	1.29E-03	1.67E+04
dme-mir-4969	-3.94E-01	3.68E+00	4.04E+00	4.45E-02	1.02E-01	4.95E-07	2.14E+02
dme-mir-13b-2	-6.09E-02	1.21E+01	4.00E+00	4.55E-02	1.03E-01	1.37E-03	8.44E+04
dme-mir-994	7.58E-01	2.15E+00	3.94E+00	4.73E-02	1.06E-01	5.78E-05	5.60E+01
dme-mir-2a-1	6.11E-02	9.73E+00	3.75E+00	5.27E-02	1.17E-01	7.63E-04	1.63E+04
dme-mir-304	-6.66E-02	1.08E+01	3.65E+00	5.61E-02	1.23E-01	1.61E-03	3.35E+04
dme-mir-4913	-1.18E+00	1.25E+00	3.25E+00	7.16E-02	1.54E-01	2.34E-06	1.70E+01
dme-mir-965	-1.62E-01	5.85E+00	3.24E+00	7.19E-02	1.54E-01	4.51E-04	1.07E+03
dme-mir-4984	-1.17E+00	1.25E+00	3.15E+00	7.57E-02	1.61E-01	2.34E-06	1.70E+01
dme-mir-1005	-2.23E-01	4.78E+00	2.98E+00	8.41E-02	1.77E-01	1.19E-04	4.98E+02
dme-mir-10	-5.54E-02	1.08E+01	2.90E+00	8.85E-02	1.84E-01	1.34E-03	3.41E+04
dme-mir-4950	1.71E+00	6.62E-01	2.72E+00	9.91E-02	2.04E-01	2.01E-04	2.00E+00
dme-mir-1017	-1.46E-01	5.68E+00	2.36E+00	1.25E-01	2.54E-01	3.77E-04	9.47E+02
dme-mir-9c	-5.15E-02	1.17E+01	2.28E+00	1.31E-01	2.64E-01	1.72E-03	6.35E+04
dme-mir-1004	-7.95E-02	7.58E+00	2.23E+00	1.35E-01	2.70E-01	8.13E-04	3.56E+03
dme-mir-4940	-4.76E-01	2.45E+00	2.11E+00	1.46E-01	2.87E-01	1.35E-04	7.50E+01
dme-mir-275	4.38E-02	1.04E+01	2.10E+00	1.47E-01	2.87E-01	9.51E-04	2.59E+04
dme-mir-971	-1.54E-01	5.35E+00	2.09E+00	1.48E-01	2.87E-01	2.80E-04	7.49E+02
dme-mir-972	5.64E-01	2.05E+00	2.03E+00	1.54E-01	2.97E-01	4.13E-04	5.10E+01
dme-mir-2a-2	4.01E-02	1.05E+01	1.86E+00	1.73E-01	3.29E-01	9.32E-04	2.80E+04
dme-mir-6-3	1.35E+00	8.00E-01	1.82E+00	1.77E-01	3.34E-01	3.17E-04	5.00E+00
dme-mir-4956	1.32E+00	8.02E-01	1.76E+00	1.84E-01	3.43E-01	3.15E-06	5.00E+00
dme-mir-137	-6.37E-02	8.27E+00	1.76E+00	1.85E-01	3.43E-01	1.45E-03	5.85E+03
dme-mir-4975	-9.60E-01	1.08E+00	1.68E+00	1.95E-01	3.58E-01	2.78E-06	1.20E+01
dme-mir-1009	-1.65E-01	4.81E+00	1.64E+00	2.01E-01	3.66E-01	1.23E-04	5.02E+02
dme-mir-4962	-1.07E+00	8.86E-01	1.44E+00	2.29E-01	4.10E-01	3.15E-06	7.00E+00
dme-mir-4960	-3.45E-01	2.73E+00	1.44E+00	2.30E-01	4.10E-01	7.81E-08	9.80E+01
dme-mir-92a	8.22E-02	6.75E+00	1.43E+00	2.31E-01	4.10E-01	7.94E-04	2.03E+03
dme-mir-87	-4.81E-02	8.59E+00	1.42E+00	2.34E-01	4.11E-01	7.29E-04	7.10E+03

dme-mir-990	1.35E-01	5.03E+00	1.30E+00	2.54E-01	4.43E-01	2.12E-04	5.99E+02
dme-bantam	-3.79E-02	1.45E+01	1.28E+00	2.57E-01	4.45E-01	1.84E-03	4.23E+05
dme-mir-92b	-1.35E-01	4.95E+00	1.21E+00	2.71E-01	4.64E-01	2.10E-04	5.56E+02
dme-mir-4971	-1.08E+00	7.56E-01	1.12E+00	2.90E-01	4.95E-01	2.01E-04	4.00E+00
dme-mir-929	-6.66E-02	6.95E+00	1.10E+00	2.94E-01	4.96E-01	7.23E-04	2.34E+03
dme-mir-1008	1.58E-01	4.22E+00	9.90E-01	3.20E-01	5.36E-01	8.54E-05	3.29E+02
dme-mir-9b	3.59E-02	9.42E+00	9.50E-01	3.30E-01	5.48E-01	1.17E-03	1.31E+04
dme-mir-281-2	-3.17E-02	1.01E+01	9.02E-01	3.42E-01	5.64E-01	1.16E-03	1.98E+04
dme-mir-1007	-9.51E-02	5.50E+00	8.81E-01	3.48E-01	5.69E-01	3.93E-04	8.29E+02
dme-mir-13a	-4.56E-02	8.04E+00	8.11E-01	3.68E-01	5.96E-01	1.36E-03	5.08E+03
dme-mir-4961	4.88E-01	1.48E+00	8.03E-01	3.70E-01	5.96E-01	1.77E-06	2.50E+01
dme-mir-2b-1	-2.67E-02	1.12E+01	7.89E-01	3.74E-01	5.98E-01	1.18E-03	4.40E+04
dme-mir-4976	2.94E-01	2.36E+00	7.57E-01	3.84E-01	6.09E-01	1.93E-04	7.00E+01
dme-mir-282	3.64E-02	8.84E+00	7.26E-01	3.94E-01	6.17E-01	1.35E-03	8.83E+03
dme-mir-2283	6.48E-01	1.04E+00	7.22E-01	3.95E-01	6.17E-01	1.72E-03	1.10E+01
dme-mir-2b-2	-2.54E-02	1.14E+01	7.05E-01	4.01E-01	6.21E-01	1.25E-03	5.19E+04
dme-mir-4918	7.67E-01	8.42E-01	6.86E-01	4.07E-01	6.26E-01	3.20E-04	6.00E+00
dme-mir-2535b	-1.22E-01	4.32E+00	6.30E-01	4.27E-01	6.52E-01	9.67E-05	3.52E+02
dme-mir-1011	-1.72E-01	3.37E+00	6.16E-01	4.32E-01	6.55E-01	1.68E-07	1.71E+02
dme-mir-4983	-6.55E-01	9.28E-01	6.02E-01	4.38E-01	6.58E-01	3.15E-06	8.00E+00
dme-mir-3641	6.79E-01	8.43E-01	5.38E-01	4.63E-01	6.92E-01	3.15E-06	6.00E+00
dme-mir-4947	5.95E-01	9.23E-01	4.96E-01	4.81E-01	7.06E-01	3.01E-04	8.00E+00
dme-mir-4942	5.95E-01	9.22E-01	4.94E-01	4.82E-01	7.06E-01	3.47E-04	8.00E+00
dme-mir-4949	-5.45E-01	1.00E+00	4.86E-01	4.86E-01	7.06E-01	3.01E-06	1.00E+01
dme-mir-4909	-5.44E-01	1.00E+00	4.84E-01	4.86E-01	7.06E-01	1.69E-03	1.00E+01
dme-mir-4957	-5.31E-01	1.00E+00	4.62E-01	4.97E-01	7.16E-01	8.38E-05	1.00E+01
dme-mir-1016	-1.84E-01	2.79E+00	4.34E-01	5.10E-01	7.30E-01	5.38E-08	1.03E+02
dme-mir-8	-1.99E-02	1.50E+01	4.19E-01	5.17E-01	7.35E-01	1.57E-03	5.93E+05
dme-mir-2500	-3.05E-01	1.70E+00	4.05E-01	5.24E-01	7.40E-01	7.97E-04	3.30E+01
dme-mir-954	-5.08E-02	6.07E+00	3.74E-01	5.41E-01	7.52E-01	5.15E-04	1.27E+03
dme-mir-4987	6.37E-01	7.08E-01	3.61E-01	5.48E-01	7.52E-01	2.01E-04	3.00E+00
dme-mir-1013	-6.16E-02	5.46E+00	3.61E-01	5.48E-01	7.52E-01	3.54E-04	8.06E+02
dme-mir-4980	-6.32E-01	7.09E-01	3.58E-01	5.50E-01	7.52E-01	2.01E-04	3.00E+00
dme-mir-4910	1.88E-01	2.49E+00	3.53E-01	5.53E-01	7.52E-01	1.37E-04	8.00E+01
dme-mir-12	-1.75E-02	1.10E+01	3.49E-01	5.55E-01	7.52E-01	1.09E-03	3.78E+04
dme-mir-6-1	-6.17E-01	7.10E-01	3.41E-01	5.59E-01	7.53E-01	2.01E-04	3.00E+00
dme-mir-286	9.58E-02	4.07E+00	3.28E-01	5.67E-01	7.58E-01	4.65E-05	2.95E+02
dme-mir-4985	4.32E-01	1.00E+00	3.04E-01	5.81E-01	7.73E-01	1.84E-05	1.00E+01
dme-mir-2489	1.94E-01	2.19E+00	2.82E-01	5.96E-01	7.82E-01	2.88E-04	5.90E+01
dme-mir-4955	5.57E-01	7.11E-01	2.77E-01	5.99E-01	7.82E-01	2.01E-04	3.00E+00
dme-mir-2493	-3.12E-01	1.34E+00	2.75E-01	6.00E-01	7.82E-01	1.30E-05	2.00E+01
dme-mir-281-1	2.58E-02	8.01E+00	2.63E-01	6.08E-01	7.88E-01	1.31E-03	4.83E+03
dme-mir-955	2.84E-01	1.43E+00	2.57E-01	6.13E-01	7.89E-01	1.89E-06	2.30E+01
dme-mir-967	-2.28E-01	1.76E+00	2.48E-01	6.19E-01	7.92E-01	1.21E-06	3.60E+01
dme-mir-307b	-2.43E-01	1.56E+00	2.23E-01	6.37E-01	8.10E-01	9.47E-04	2.80E+01
dme-mir-3645	-4.37E-01	7.99E-01	2.08E-01	6.49E-01	8.19E-01	3.16E-04	5.00E+00
dme-mir-4968	-1.50E-01	2.40E+00	2.04E-01	6.52E-01	8.19E-01	1.79E-04	7.20E+01
dme-mir-4973	-2.27E-01	1.56E+00	1.94E-01	6.59E-01	8.23E-01	9.52E-04	2.80E+01
dme-mir-2279	7.43E-02	3.84E+00	1.62E-01	6.88E-01	8.53E-01	4.84E-05	2.41E+02
dme-mir-309	3.42E-01	8.83E-01	1.52E-01	6.96E-01	8.59E-01	3.39E-04	7.00E+00
